# What would it cost to scale-up private sector engagement efforts for tuberculosis care? Evidence from three pilot programs in India

**DOI:** 10.1371/journal.pone.0214928

**Published:** 2019-06-05

**Authors:** Sarang Deo, Pankaj Jindal, Devesh Gupta, Sunil Khaparde, Kiran Rade, Kuldeep Singh Sachdeva, Bhavin Vadera, Daksha Shah, Kamlesh Patel, Paresh Dave, Rishabh Chopra, Nita Jha, Sirisha Papineni, Shibu Vijayan, Puneet Dewan

**Affiliations:** 1 Indian School of Business, Hyderabad, India; 2 UCLA Anderson School of Management, Los Angeles, CA, United States of America; 3 Central TB Division, New Delhi, India; 4 Mumbai Mission for TB Control, Mumbai, India; 5 RNTCP, Gandhinagar, Gujarat, India; 6 PATH, Connaught Place, New Delhi, India; 7 World Health Partners, Patna, India; 8 Harvard Medical School, Boston, MA, United States of America; 9 Independent Consultant, Seattle, WA, United States of America; University of Hong Kong, HONG KONG

## Abstract

**Background:**

Private providers dominate health care in India and provide most tuberculosis (TB) care. Yet efforts to engage private providers were viewed as unsustainably expensive. Three private provider engagement pilots were implemented in Patna, Mumbai and Mehsana in 2014 based on the recommendations in the National Strategic Plan for TB Control, 2012–17. These pilots sought to improve diagnosis and treatment of TB and increase case notifications by offering free drugs and diagnostics for patients who sought care among private providers, and monetary incentives for providers in one of the pilots. As these pilots demonstrated much higher levels of effectiveness than previously documented, we sought to understand program implementation costs and predict costs for their national scale-up.

**Methods and findings:**

We developed a common cost structure across these three pilots comprising fixed and variable cost components. We conducted a retrospective, activity-based costing analysis using programmatic data and qualitative interviews with the respective program managers. We estimated the average recurring costs per TB case at different levels of program scale for the three pilots. We used these cost estimates to calculate the budget required for a national scale up of such pilots. The average cost per privately-notified TB case for Patna, Mumbai and Mehsana was estimated to be US$95, US$110 and US$50, respectively, in May 2016 when these pilots were estimated to cover 50%, 36% and 100% of the total private TB patients, respectively. For Patna and Mumbai pilots, the average cost per case at full scale, i.e. 100% coverage of private TB patients, was projected to be US$91 and US$101, respectively. In comparison, the national TB program’s budget for 2015 averages out to $150 per notified TB case. The total annual additional budget for a national scale up of these pilots was estimated to be US$267 million.

**Conclusions:**

As India seeks to eliminate TB, extensive national engagement of private providers will be required. The cost per privately-notified TB case from these pilots is comparable to that already being spent by the public sector and to the projected cost per privately-notified TB case required to achieve national scale-up of these pilots. With additional funds expected to execute against national TB elimination commitments, the scale-up costs of these operationally viable and effective private provider engagement pilots are likely to be financially viable.

## Background

India bears the largest portion of tuberculosis (TB) disease burden in the world in terms of incidence, prevalence, and mortality [1]. Of the 2.6 to 6.8 million estimated cases in India in 2014, 1.2 to 5.3 million cases (46–79%) were estimated to be treated in the private sector (either for-profit or not-for-profit non-government providers), but only 0.2 million (4–16%) of these were notified [2, 3]. TB diagnostic and treatment practices by private providers have been repeatedly found to be far short of the national standard guidelines [4–7]. Several Public-Private Mix (PPM) models, aimed at promoting standard TB care practices and encouraging referral of diagnosed TB cases to the public sector among private providers, have been implemented by the Revised National TB Control Program (RNTCP) [7–11]. These models contributed only 0.5%–2.5% of the estimated cases in the private sector in 2014 [1, 12–15]. It has been conjectured that public sector activities have been prioritized over private provider engagement, leading to a low uptake of any models or schemes for private provider engagement [15–17]. Moreover, providers who are aware of PPM models may limit their involvement due to restrictions on prescription of TB treatment [18], from fear of losing their revenues to the public sector through patient referrals [13, 14, 16, 18, 19], or from concerns about the quality and access of care available to patients at public facilities [20].

The National Strategic Plan (NSP) for TB Control, 2012–2017 proposed the development of interfacing agencies to engage with providers in order to overcome these challenges and scale up private sector engagement efforts [21, 22]. The plan recommended that, instead of promoting referrals as in the existing PPM models, these interfacing agencies should encourage TB case notifications by providing subsidized (or free) diagnosis and treatment to patients treated by private providers if they followed Indian standards of TB care. To test the feasibility of a model based on these broad principles, in 2014 the RNTCP initiated a series of three pilots, labeled Universal Access to TB Care (UATBC). In the large cities of Mumbai (Maharashtra) and Patna (Bihar), the RNTCP utilized the services of a Private Provider Support Agency (PPSA) to engage providers, deploy diagnostic and treatment services, and support patient adherence. In parallel, the RNTCP deployed a similar but lower intensity pilot in the rural district of Mehsana (Gujarat), which had far fewer private providers, where RNTCP staff members themselves fulfilled the interfacing function. A critical success factor to ensure the scale-up of such pilots and their successful integration into RNTCP is to ensure that their cost is not significantly higher than the cost of providing TB care in the public sector, as is typically assumed. In this paper, we address this issue and estimate the operating costs of these pilots at various levels of population coverage and estimate the budget required to scale them at a national level.

## Methods

### Study setting

A comprehensive description of the pilots is available in a report by Ministry of Health and Family Welfare [23]. Briefly, the pilots in Mumbai and Patna involved contracted Private Provider Support Agencies (PPSA) who, on behalf of RNTCP, engaged with formal and informal private providers through visits by full-time trained field officers, continuing medical education seminars and training workshops. They provided free drugs, subsidized diagnostic tests (chest X-ray and GeneXpert) to patients of engaged providers with the help of an integrated information and communications technology (ICT) platform, including a call center that generated and validated electronic vouchers. They also provided treatment monitoring and adherence support services to patients through a combination of periodic household visits by field officers and calls by call center agents. Modest monetary incentives were provided to the chemists who provided free drugs to patients against the electronic vouchers. In addition to these standard components, there were a few differences across pilots to allow implementing agencies to customize their intervention to the local context. The Patna pilot included patient subsidies for Sputum Smear Microscopy and provider incentives for ordering diagnostic tests and initiating patients on treatment. The pilot in Mehsana did not involve any PPSA or employ additional full-time field officers (beyond the existing RNTCP staff in the district) and did not provide any patient subsidy for diagnostic tests. Incremental costs for the pilots and associated technical assistance were borne by RNTCP’s development partners, including the Bill & Melinda Gates Foundation, USAID, and the Global Fund. As of September 2016, Patna, Mumbai, and Mehsana pilots, had engaged with 927, 3670, and 319 providers, had 8648, 6881, and 1414 patients under treatment and had notified 35284, 32915 and 6684 cases, respectively, since their inception [23]. An overview of the program characteristics of the three pilots and their scale is provided in Table 1 and Table 2.

**Table 1:** Scale of pilots in September 2016.

	Patna	Mumbai	Mehsana
Engaged providers	927	3670	319
Patients currently on treatment in this month	8648	6881	1414
Patients initiated on treatment in this month	1356	1371	269
GX tests ordered	746	1207	Not applicable

**Table 2:** Characteristics of the intervention.

Patna	Mumbai	Mehsana
Urban PPSA	Urban PPSA	Rural–RNTCP run
More incentives	No incentives	Less incentives
Less NGO staff	More NGO Staff	No NGO staff
Less GX use	More GX use	No GX
Free drugs	Free drugs	Free drugs

### Study design

We used quantitative and qualitative programmatic data of the three pilots from their respective launches (July to September 2014) until May 2016 to conduct a retrospective activity-based costing analysis [24]. We included recurring monthly expenses of each model in our analysis and excluded the initial set-up costs.

### Data

**Fig 1:**
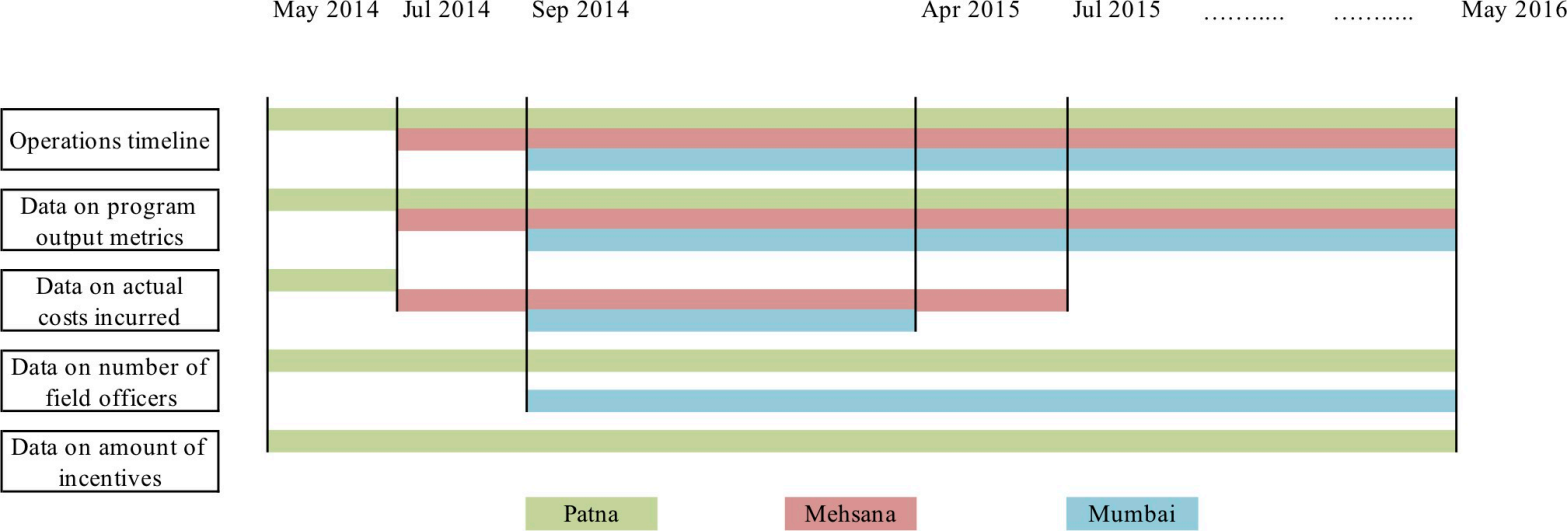
Timelines for PPSA operations and for data availability.

We collected program data from three sources. First, we accessed monthly performance reports submitted by the three pilots to BMGF in a common format. These included various program output metrics such as the numbers of providers mapped and engaged, patients initiated on treatment, patients currently on treatment, diagnostic tests (GeneXpert, chest X-ray, and Sputum Smear Microscopy) conducted and total patient-months of drugs sold in the private sector as reported by IMS Health (3). Second, we collected data on actual costs incurred for various components such as salaries of office staff and field officers and administrative costs (e.g., audit costs, travel costs) over different time periods at the beginning of these pilots (Fig 1). For Mumbai, these data were available for eight months from September 2014 till April 2015. For Patna, only a monthly estimate was available. For Mehsana, these costs were available for the first year of operation, from July 2014 to June 2015. We also obtained monthly data on the number of field officers in Mumbai and Patna pilots and the amount of incentives provided in the Patna pilot for the entire duration of operations (Fig 1). Third, we conducted unstructured interviews with program managers of the three pilots to identify relationships between program inputs and outputs (e.g., number of field officers and number of engaged providers), to identify program activities that acted as cost drivers and to understand how they impacted the costs.

### Analysis

#### Classification of cost components

We developed a common cost structure across all three pilots comprising nine components, each with multiple subcomponents (Table 3). Based on expert judgment of the program managers, we classified the cost subcomponents that varied with program scale (as measured by the number of patients or number of providers) as variable costs and the rest as fixed costs. The former included salaries of field staff, diagnostic and treatment subsidy, costs for incentives, call center agents and other ICT costs. The latter included salaries of office staff, costs for provider sensitization, community outreach costs, and administrative costs. We labeled the direct costs related to drugs and diagnostic services as commodity costs and the rest as programmatic costs.

**Table 3:** Cost structure and sources of data.

Cost Category	Nature*	Driving Activity	Unit cost Average cost per month (in US$)			Source of unit cost**
			Patna	Mumbai	Mehsana	
**1. Staff Costs**						
Salary of PPSA Office Staff	F		$15,077.0	$28,930.0		2
Field expenses of office staff	F			$1,000.0	$32.1	2
Out of town expenses of office staff	F			$1,923.1		2
Salary of Lab Staff	F		$646.0			2
Salary of contracted staff (field)	V	Field Officers	$296.7	$516.72		Patna: 3; Mumbai: Derived using 1 and 2
		Monitoring Officers	$325.1			
		Project Coordinators	$265.2			
		Area Managers	$484.9			
Salary of CBO office staff	F			$4,243.0		2
Salary of CBO field staff	V	CBO Staff		$240.4		Derived using 1, 2 and 3
Salary of CBO SCT staff	V	Sample Transport	$0.5			Patna: 3; Mumbai: Derived using 1, 2 and 3
		Field Officers for Sample Collection	$184.6	$115.4		
**2. Provider training & sensitization**						
Provider training workshops	V	Formal Providers	$30.8			Patna: 3; Mumbai, Mehsana: 2
	F			$1,418.8	$109.0	
**3. Lab Operations**						
Lab Consumables	V	Lab Reagents	$0.3			Derived using 1 and 3
GeneXpert Maintenance cost	F		$192.0			2
**4. Diagnosis**						
X ray subsidy	V	X rays	$3.9	$3.2		Patna: 3; Mumbai: Derived using 1 and 2
GX subsidy	V	GX Tests	$18.5	$18.8		
Sputum Test subsidy	V	Sputum test	$3.1			
**5. Treatment**						
Drugs cost/subsidy	V	Treatment vouchers	$7.4	$6.9	$6.9	3
**6. ICT Costs**						
Call center seats	V	Call Centre Agents	$215.4	$2,132.3	$2,132.3	2
SMS costs	V			$0.01	$0.01	3
Telecom costs	V	Call Minutes	$0.02	$0.02	$0.02	3
IT Resource Cost	F				$92.3	2
Operational Costs	F		$3,338.0			2
**7. Community Outreach Costs**						
IEC activities	F			$922.2		2
**8. Other Administrative Costs**						
Printing costs	F		$36.0	$102.5		Patna: 3; Mumbai: 2
Facilities Cost	F			$5,384.6		2
Audit costs	F			$591.7	$1,282.1	2
CBO office supplies & miscellaneous	F			$4,191.7		2
**9. Incentives**						
Diagnostic Incentives	V	Diagnostic Incentives (3 types)	$0.8-$3.1			2
Treatment Incentives	V	Treatment Incentives (5 types)	$1.5-$3.1		$0.21	Patna: 2; Mehsana: 3

Note

* F = Fixed, V = Variable.

** Monthly performance reports = 1, Actual costs data = 2, Program manager interviews = 3

#### Retrospective estimation of actual costs

For fixed costs, we calculated an average of the actual monthly costs incurred over eight months from September 2014 to April 2015 for Mumbai, and twelve months from July 2014 to June 2015 for Mehsana, and considered the monthly estimate for Patna (Fig 1) to use as estimates up to May 2016. For variable costs, we estimated the magnitude of cost drivers identified by program managers, either from directly available monthly program data (e.g., number of field staff, diagnostic and treatment vouchers) or based on their relationship with other program activities for which data were available. For instance, we estimated the number of sputum collection and transportation agents based on the number of sputum samples, and the number of call center agents based on the number of treatment and diagnosis vouchers issued. We obtained unit costs for these cost drivers (e.g. salary of a call center agent, sputum collection and transportation agent, telecommunication costs per SMS or per minute) from our data sources. Where such data were not available, we derived the unit costs from actual costs incurred and the scale of the cost driving activities over the same period (e.g. salary of field staff, cost of lab consumables). We combined the unit cost estimates and the monthly scale of these cost driving activities to estimate the monthly variable costs up to May 2016. We combined the monthly fixed and variable cost estimates with the number of patients on treatment in each month to estimate the average cost per case per month. We scaled this estimate by the average duration of treatment (calculated as the moving average of the ratio of number of patients on treatment and number of patients initiated on treatment) to estimate the average cost per case.

#### Prospective cost projections

**Fig 2:**
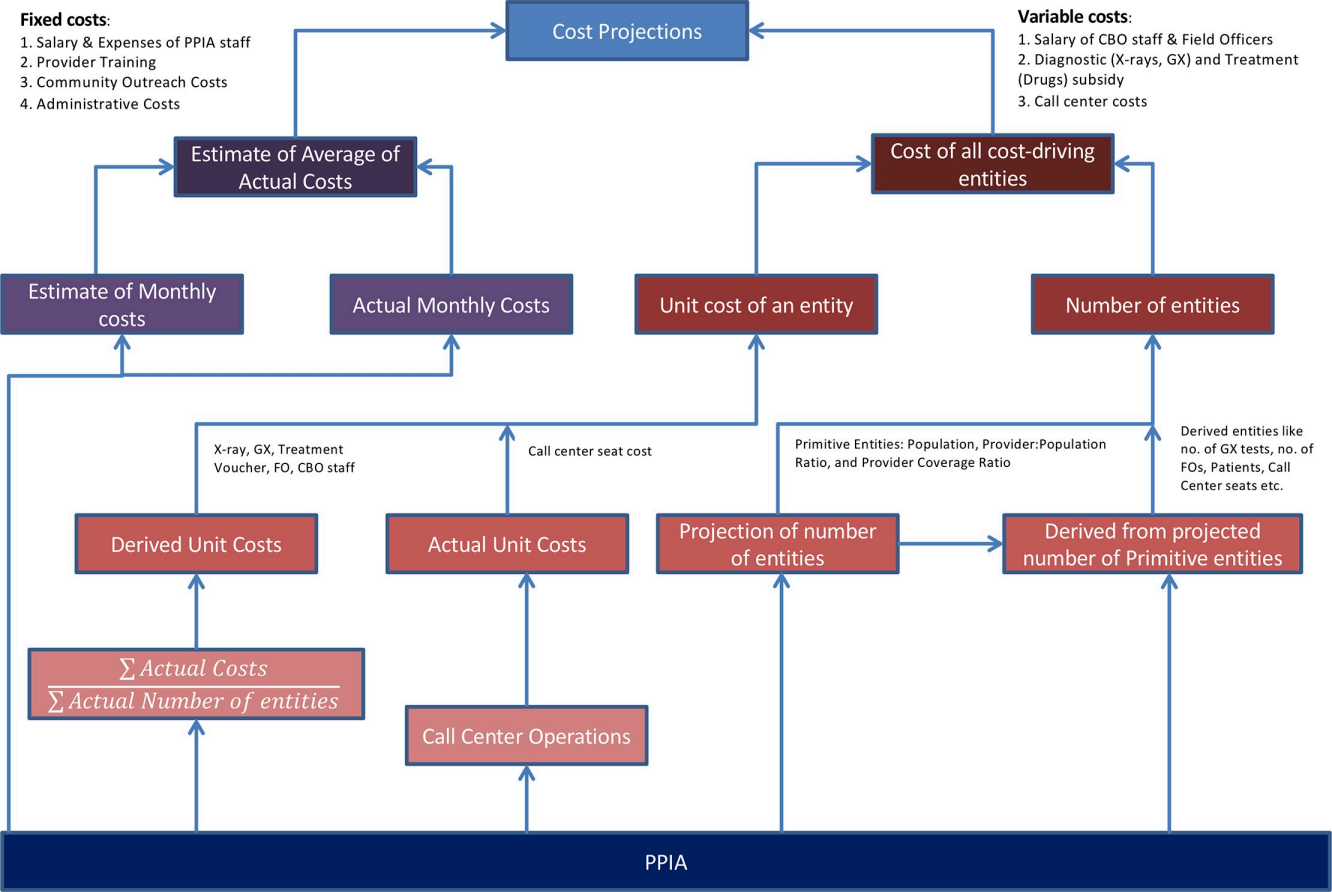
Schematic representation of the methodology for prospective cost estimation.

Fig 2 provides a pictorial representation of our methodology for prospective cost projections for the period after May 2016. First, we estimated the relationship between the number of engaged providers and the magnitude of other key program outputs (e.g. number of treatment initiations, number of patients under treatment and number of diagnostic vouchers) and the relationship between these program outputs and the respective cost drivers (e.g. number of field officers, call center agents) based on retrospective program data (see S1 Table and S1 Text for details). Then, we used these relationships along with an assumed rate of scale-up of engaged providers to project the magnitude of the respective cost drivers. We multiplied the previously estimated unit costs with the projected magnitude of respective cost drivers to obtain projections for the variable cost subcomponents. We assumed that the fixed cost subcomponents remain unchanged after May 2016. We calculated the average cost per case per month and average cost per case using the method employed for the retrospective estimation as described above. We also calculated monthly population coverage of the program based on the projected number of patients on treatment in that month and patient-months of drugs sold in the private sector which itself was assumed to be constant after May 2016 [3].

#### Budget implications

We allocated patient-months of drugs sold in the private sector in each state (3) into urban and rural categories using the average annual risk of tuberculosis infection in the urban and rural areas [25] and each state’s population in these areas. In accordance with current practice, we categorized commodity costs under national TB program budget and programmatic costs under respective state budgets. We applied estimates of the average costs per case per month (at 100% population coverage) from Mumbai and Patna models to obtain budget estimates for urban areas and those from the Mehsana model for rural areas.

## Results

**Fig 3:**
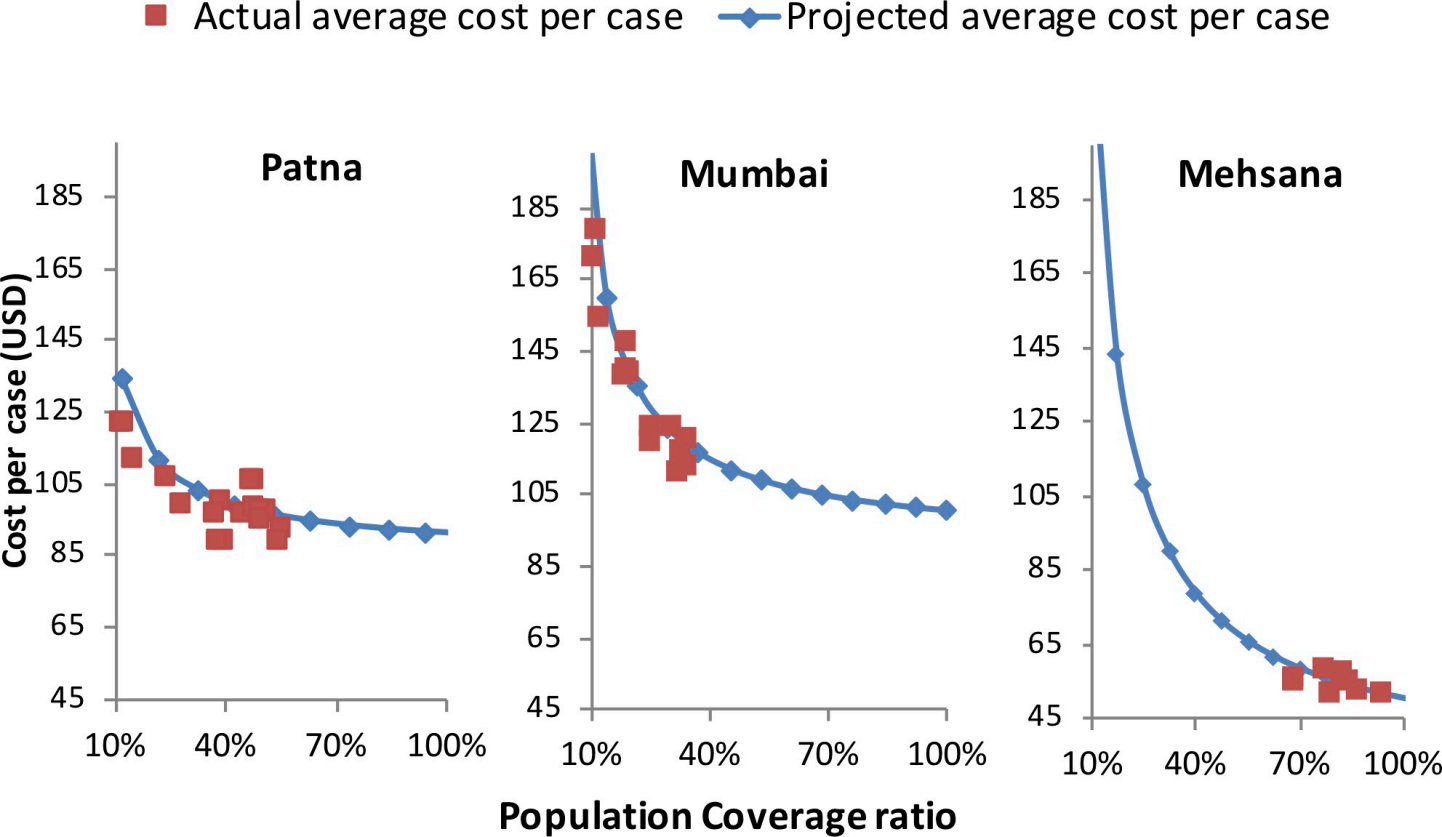
Actual and projected costs per case for Patna, Mumbai and Meshana.

Fig 3 displays retrospective estimates as well as prospective projections of the average cost per case in Patna, Mumbai and Mehsana. In May 2016, the estimated average cost per case was US$95 and US$110 for Patna and Mumbai pilots at population coverage ratios of 50% and 36%, respectively. For Mehsana pilot, which provided fewer diagnostic services and had already reached close to 100% population coverage ratio in May 2016, the average cost per case was estimated to be US$50. For Patna and Mumbai pilots, the average cost per case at 100% population coverage was estimated to be US$91 and US$101, respectively. At 100% coverage, the commodity cost per case was estimated to be US$58, US$67 and US$30 whereas the programmatic cost per case was estimated to be US$33, US$34 and US$21 for Patna, Mumbai and Mehsana, respectively. The largest component of cost was commodity cost (drugs and diagnostics for Patna and Mumbai, drugs for Mehsana) followed by field staff (for Patna and Mumbai) and ICT costs.

The significantly lower cost of the Mehsana pilot was due to absence of field staff (beyond two full time RNTCP employees) and diagnostic subsidies. Within the urban pilots, Mumbai pilot did not provide monetary incentives to providers but had higher diagnostic costs compared to Patna (US$32.3 vs. US$20.8) due to greater uptake of GeneXpert by engaged providers. Mumbai pilot also had higher office staff cost (US$7.1 vs. US$4.7) and ICT costs (US$11.8 vs. US$9.6) compared to Patna pilot (Table 4). The estimated average cost per case decreased for all three pilots with increased population coverage as the fixed costs were spread over a larger program (both in terms of number of providers and number of patients). This decline was much steeper for the Mumbai pilot because of its higher fixed cost structure compared to Patna and Mehsana pilots.

**Table 4:** Average cost per case at full scale.

Average cost per case (US$)	Patna	Mumbai	Mehsana
Office Staff Cost	4.67	7.11	0.10
ICT costs	9.64	11.75	15.32
Field Staff Cost	12.06	12.88	0.00
Incentives Cost	5.26	0.00	0.89
Other Costs	1.44	2.48	4.49
*Programmatic Cost*	33.07	34.23	20.80
Diagnostic Cost	20.79	32.34	0.00
Treatment Cost	37.31	34.78	29.52
*Commodity Cost*	58.09	67.12	29.52
***Total Cost***	**91.16**	**101.35**	**50.32**

The national budget for scaling such engagement programs in urban and rural areas at the national level was estimated to be US$119 million and US$148 million, respectively. Table 5 provides state wise budget estimation along with the actual state wise allocation of funds for 2014–15 under RNTCP.

**Table 5:** State wise budget for full-scale implementation of the interventions.

State	Annual Budget (mn USD)			Funds allocated in 2014–15 under RNTCP (mn USD)
	Rural	Urban	Total	
Andhra Pradesh	2.50	2.93	5.42	4.14
Assam & North East	1.33	0.69	2.02	4.71
Bihar	6.29	1.87	8.16	3.77
Chhattisgarh	0.86	0.61	1.46	1.59
Delhi	0.08	7.33	7.41	2.12
Goa	0.02	0.09	0.12	0.15
Gujarat	2.11	3.66	5.76	3.01
Haryana	0.91	1.13	2.03	1.34
Himachal Pradesh	0.22	0.06	0.28	0.77
Jammu & Kashmir	0.40	0.35	0.74	1.25
Jharkhand	1.20	0.88	2.08	1.93
Karnataka	1.32	1.93	3.25	3.46
Kerala	0.33	0.71	1.04	1.70
Madhya Pradesh	2.99	2.66	5.65	3.60
Maharashtra	3.31	6.35	9.66	8.47
Orissa	0.51	0.24	0.75	2.21
Punjab	0.93	1.43	2.36	1.60
Rajasthan	3.25	2.51	5.76	2.72
Tamil Nadu	1.16	2.56	3.72	3.54
Uttar Pradesh	16.57	11.06	27.63	8.60
Uttaranchal	0.93	0.95	1.88	0.76
West Bengal	1.28	1.39	2.68	4.09

Note: Andhra Pradesh includes Telangana; North East includes Arunachal Pradesh, Manipur, Meghalaya, Mizoram, Nagaland, and Tripura; Gujarat includes Gujarat and Daman & Diu; Kerala includes Kerala and Lakshadweep; Maharashtra includes Maharashtra and Dadar and Nagar Haveli; Punjab includes Punjab and Chandigarh; Tamil Nadu includes Tamil Nadu, Pondicherry, and Andaman & Nicobar; West Bengal includes West Bengal and Sikkim.

## Discussion and conclusions

Innovative models, such as interfacing agencies, have been proposed as a potential mechanism for large scale private sector engagement for TB diagnosis and treatment in India. In this paper, we take the first step towards obtaining a realistic estimate of the budget required for a successful national scale up of such models. Towards this end, we used a detailed programmatic understanding to conduct a cost analysis of the three UATBC pilots implemented in Patna, Mumbai and Mehsana. Our results suggest that, at full scale, i.e., 100% population coverage, the average recurring cost per case would be between US$90 and US$100 for urban pilots (e.g. Mumbai and Patna) and around US$50 for rural pilots (e.g. Mehsana).

Costs of Mumbai and Patna pilots are significantly higher than the Mehsana pilot. The main drivers for this difference were the diagnostics and staffing costs, which were subsumed in the existing RNTCP budget for Mehsana and hence were not accounted for in the pilot. ICT costs were slightly higher for Mehsana on average because of its smaller scale of operations. Diagnostic cost was lower for Patna than Mumbai because of the use of smear microscopy and lower uptake of GX. Notably, Patna pilot included a referral incentive for providers, but it only accounted for around 5% of the overall average cost. Comparison of Patna and Mumbai pilots highlight the possibility of allowing implementing agencies to tailor their intervention to the local context yet achieve comparable costs which, in turn, makes it possible to develop output-based contracts without considerable cost escalation.

These cost estimates are well within with the budgeted cost of US$150 per TB case in the public sector in India in 2014 [26, 27]. Moreover, given that roughly half of the TB cases in India are estimated to be treated in the private sector, the estimated budget requirement for national scale up of these private sector engagement pilots is comparable to the national TB program budget of US$252 million in 2014. Simply put, it is unsurprising that detecting and treating twice as many TB patients may be expected to cost roughly twice as much budget. It may be welcome news that doing so among private providers is achievable at a similar cost as in the public sector, given the broad dominance of private sector health care delivery in India. Furthermore, our state-wise budget estimates are comparable with the funds allocated to states under RNTCP in 2014–15 with a few exceptions. These comparisons provide strong evidence that scaling up private sector engagement efforts at national level is financially viable given pre-existing willingness to spend on TB care in India.

Comparison of cost estimates presented in this study with prior experiences must be made judiciously, as the effectiveness of the interventions studied here are very different from that of previously studied PPM models. Previous studies analyzing PPM models in Kannur, Bangalore, Hyderabad and Delhi have calculated average costs in different ways and have reported much lower cost estimates—in the range of US$25 to US$69 [7, 10, 28]. The higher cost structure of the current private sector engagement pilots compared to the earlier PPM models may be attributed to wide differences in the scope and scale of program activities and reflect the threshold needed for higher effectiveness. Between January 2015 and May 2016, the UATBC pilots in Mumbai, Patna, and Mehsana were responsible for 36%, 82% and 58%, respectively, of all TB notifications [29] compared to less than 15% of private sector notifications contributed by all PPM efforts combined in 2015 [1, 15]. Arguably, the more comprehensive scope of activities is the key driver for much larger case notification rates in the current private sector engagement pilots compared to the earlier PPM models. With high levels of coverage and effectiveness required to achieve national TB control targets, prior experiences with limited effectiveness and their associated low costs should act as a warning signal against under-provision of services and underinvestment in private engagement.

Our study has certain limitations. We used retrospective programmatic data for all cost driving activities and relied on qualitative insights of program managers wherever such data were not available. Furthermore, data on the actual costs used to estimate monthly fixed costs were available only for a limited period. Our assumption that these fixed costs would remain unchanged at higher scale of operations might not hold true for all cost subcomponents. For budget estimation, we assumed that for other rural and urban areas in the country would be similar to the average cost per case for one rural area and two urban areas, where these pilots were implemented. However, the actual costs might vary slightly depending on various socio-economic and demographic factors. We also assumed that the average cost per case per month will remain constant through the scale-up. However, our study was limited to short-term programmatic cost of implementing these engagement pilots, to inform policy makers on short term budget implications that appear to be required to approach the government’s ambitious targets. We did not include the impact of these pilots on patient costs (either increase or decrease) and potential cost saving due to the effectiveness of these pilots in averting future TB cases. Future studies could augment our cost estimates with patient costs and impact on disease transmission to conduct a comprehensive cost-effectiveness analysis.

## Supporting information

S1 Table Relationships between cost driving activities and program outputs. (DOCX)

S1 Text Scale of driving activities. (DOCX)
