# Supplementary material for: What would it cost to scale-up private sector engagement efforts for tuberculosis care? Evidence from three pilot programs in India
Source: PLoS One. 2019 Jun 5;14(6):e0214928. doi: 10.1371/journal.pone.0214928 (PMC6550378; doi:10.1371/journal.pone.0214928)
Supplement: S1 Table — (DOCX) [file pone.0214928.s001.docx]

S2 Table: Relationships between cost driving activities and program outputs

| Driving Activity | Derived from | Relationship |
| --- | --- | --- |
| Field Officers | Existing Providers, New providers, Patients on treatment | On-field time required per month / Available time per field officer per month |
| Monitoring Officers | Field Officers | Field Officers * Monitoring Officers required per field officer |
| Project Coordinators | Field Officers | Field Officers * Project Coordinators required per field officer |
| Area Managers | Project Coordinators | Project Coordinators * Area Managers required per Project Coordinator |
| CBO Staff | Formal Providers, Informal Providers, Patients on Treatment | FP * Staff required per FP + IFP * Staff required per IFP + Patients * Staff required per patient |
| Sample Transport | Patients initiated on treatment | Patients initiated on treatment * ratio of samples transported per initiated patient |
| Field Officers for Sample Collection | GeneXpert Samples | GX Tests * Samples collected per Sputum Collection Field Officer |
| Formal Providers | Practicing Providers | Practicing Providers * Provider Coverage Ratio * Ratio of FP to the Engaged Providers |
| Lab Reagents | GX Tests | Equal to the number of GX tests done |
| X rays | FPs, IFPs | FP * X-Rays ordered per FP + IFP * X-Rays ordered per IFP |
| GX Tests | FPs, IFPs | FP * GX ordered per FP + IFP * GX ordered per IFP |
| Sputum test | FPs, IFPs | FP * Sputum tests ordered per FP + IFP * Sputum tests ordered per IFP |
| Treatment vouchers | Patients currently on treatment | Equal to the number of patients currently on treatment |
| Call Centre Agents | Total Call duration required | Total calls duration required per month / Available time per call center agent |
| SMS | Patients initiated on treatment, Patients currently on treatment | New patients * SMS per new patient + Patients currently on treatment * SMS per patient currently on treatment |
| Call Minutes | GX Tests, Treatment Vouchers Generated and Validated, Patients Initiated on treatment, Patients currently on treatment | Summation of number of all call driving activities * Call minutes required per month per driving activity) |
| Incentives | Incentives, FPs, IFPs | FPs * Number of Incentives of each type per FP + IFPs * Number of incentives of each type per IFP |
| Patients Initiated on Treatment | FP, IFP, GX Tests, X-Rays | Provider of each type * Number of patients initiated per type per month GX Tests * Confirmed patients per GX test + X-Rays * Confirmed patients per X-ray |
| Patients Currently on Treatment | Patients Initiated on Treatment, Average Duration of Treatment | Patients Initiated on Treatment * Average Treatment Duration |
