# Supplementary material for: What would it cost to scale-up private sector engagement efforts for tuberculosis care? Evidence from three pilot programs in India
Source: PLoS One. 2019 Jun 5;14(6):e0214928. doi: 10.1371/journal.pone.0214928 (PMC6550378; doi:10.1371/journal.pone.0214928)
Supplement: S1 Text — (DOCX) [file pone.0214928.s002.docx]

# S1 Text: Scale of driving activities

The methodology for calculation of the scale of the driving activities mentioned in table 3 is intuitive for most cases and is briefly explained in the table itself. Some others are not so intuitive, and we describe those in this section.

## Formal Providers

We assumed that a PPSA scales up in a city by bringing in more providers under its coverage rather than increasing the number of mapped providers. We used the provider-coverage ratio, which solely drives scaling up of operations in this analysis, to derive the number of engaged providers. We then calculated the number of formal providers as a fixed ratio of engaged providers, with the ratio being derived using the operations data. We used the same approach to calculate the number of informal providers also.

The calculations of the number of providers is pretty intuitive but we explain these in slight detail as the number of providers – formal and informal – form a very essential part of the projections of scaling up of other driving activities. The future scale of all other driving activities like number of GX tests, number of field staff, patients initiated for treatment in a month etc. is derived from the number of providers either directly or indirectly. In a different context and setting, where the number of mapped providers also increases, a similar analysis can be performed to derive the projections for the number of engaged providers and consequently other cost driving activities.

## Field Officers (FO)

The projection of number of field officers is derived considering the activities a field officer performs on the ground, which is slightly different for WHP and PATH.

For WHP, FOs engage primarily in three activities: visiting newly engaged providers, visiting already engaged providers, and visiting patients currently on treatment. Duration of a single visit for each of these three activities is 20 minutes (according to our discussions with the program manager). The number of monthly visits required is 4, 1.5 and 2 respectively for new providers, existing providers, and patients currently on treatment. We calculated the required FO time per activity using the time required per visit for each type of visit and the number of such visits. We derived the total FO visit duration required per month as a sum-total of required FO time for all activities. Based on discussions with the program manager, we assumed the average visit duration available per day per field officer to be 300 minutes taking into account the travel time, and each month to be having 25 working days. Using these, we calculated the total time available per FO per month. We then derived the FOs required per month as the number of FOs that would be required to fill the total required visit duration.

For PATH, the field officers are involved primarily in visiting engaged providers and hence a simple ratio of number of providers that can be covered by one field officer has been used to derive the required number of field officers.

## CBO SCT and Field Staff

Community Business Organizations are similar to the non-profit organizations. PPSAs hire CBO employees primarily for sputum collection and transportation (SCT); we term these as CBO SCT Staff.

Using actual operations tracking data of the PPSAs, we calculated the monthly capacity of a single SCT staff member as the ratio of samples transported per staff member. We then scaled the number of required SCT staff members using the projected monthly number of samples. The projected number of samples for PATH is the projected monthly number of GX tests, whereas for WHP it depends on the number of patients initiated in a month.

For PATH, a CBO staff does additional field activities of a similar nature as done by the field officers at WHP, which we term as CBO Field Staff and forms a separate sub-component. A CBO Field Staff undertakes the following activities: Visiting patients for counselling, visiting formal and informal providers to maintain engagement.

Using actual operations data, we calculated the monthly capacity of an FO as a ratio of number of engaged providers (formal and informal), and the number of patients counselled per month per FO. We then projected the number of required CBO field staff using the projected monthly number of engaged providers and patients.

## Call center operations

Telecommunication costs are driven by call minutes in a month. We computed the total calling duration required in a month considering the set of activities that require telephone calling.

For WHP, there are primarily five activities in this set: Diagnostic Voucher Generation, Treatment Voucher Generation, Treatment Voucher Validation, Patients Registration, and Adherence Calls which are respectively driven by the number of GX tests done in a month, treatment vouchers generated and validated in a month, patients initiated, and patients currently on treatment. The activities are same for PATH excluding Patients Registration and Treatment Vouchers Validation. Based on discussions with program managers, we noted the duration per call for each of these activities. Using the projected monthly number of calls of each type, we then derived the total projected monthly call duration. We derived the number of call center agents required per month using the projected monthly call duration and the available monthly call minutes per agent.

## Incentives

WHP employs some schemes to incentivize both formal and informal providers for better diagnosis and treatment. They use five types of incentives for formal providers, and three for informal providers with fixed amount of incentives as given in table 2. We noted the actual number of incentives given out in the past using operations data from MIS. We then derived the projected number of incentives as a ratio of the projected number of providers, the ratio being the actual number of incentives per provider (formal and informal) for each kind of incentive.
